# Influence of Printing Orientation on Surface Roughness and Gloss of 3D Printed Resins for Orthodontic Devices

**DOI:** 10.3390/ma18030523

**Published:** 2025-01-23

**Authors:** Cecilia Goracci, Carlo Bosoni, Patrizia Marti, Nicola Scotti, Lorenzo Franchi, Alessandro Vichi

**Affiliations:** 1 Department of Medical Biotechnologies, University of Siena, 53100 Siena, Italy; 2 Department of Experimental and Clinical Medicine, University of Florence, 50127 Florence, Italy; carlo.bosoni@unifi.it (C.B.);; 3 Santa Chiara Fab Lab, Department of Social, Political and Cognitive Sciences, University of Siena, 53100 Siena, Italy; patrizia.marti@unisi.it; 4 Department of Surgical Sciences, Dental School Lingotto, University of Turin, 10124 Turin, Italy; 5 School of Dental, Health and Care Professions, University of Portsmouth, Portsmouth PO1 2QG, UK

**Keywords:** 3D printing, resin, aligner, printing orientation, surface roughness, surface gloss

## Abstract

The study aims to assess the effect of printing orientation on surface roughness and gloss of resins for 3D printing of aligners. Squared specimens (14 × 14 × 4 mm) were printed using Dental LT Clear (Formlabs, Somerville, MA, USA; LT) or Tera Harz TC-85 DAC (Graphy, Seoul, Republic of Korea; TC) with different orientations: 0° (horizontal), 90° (vertical), and as per the manufacturer’s recommendation (40° for LT, 60° for TC). A profilometer was used to measure roughness (Ra) in µm, while gloss was recorded in gloss units (GU) with a glossmeter. The collected data were statistically analyzed. Material type did not significantly influence roughness, while print orientation was an influential factor, with the orientation recommended by the manufacturer yielding the roughest specimens. Vertical printing resulted in significantly higher roughness than horizontal. Material type was a significant factor for gloss, with TC exhibiting significantly higher gloss than LT. Print direction significantly influenced gloss, with vertical printing resulting in the highest gloss. The finding of higher roughness for vertical prints can be explained by the presence of a greater number of layers. The superior gloss exhibited by TC regardless of print angulation could be related to the effective cleaning of uncured resin by centrifugation and to the high degree of monomer conversion in nitrogen atmosphere.

## 1. Introduction

A great interest is currently surrounding the use of resins for the direct three-dimensional (3D) printing of orthodontic aligners. It has been reported that, in comparison with the thermoforming procedure, direct printing simplifies the workflow, reduces the amount of plastic waste, and produces aligners with improved adaptation whose thickness can be better controlled [1,2,3,4].

Direct-printed aligners have been tested for fit accuracy, mechanical properties, citotoxicity, and estrogenicity [2,3]. Yet the evidence so far collected on their surface characteristics is limited [5,6,7]. Nevertheless, such properties are clinically relevant for their bearing on plaque retention, staining and translucency loss of the aligner, tongue comfort, aligner wear, and consequent monomer leaching [2,3,4,5]. Additionally, the choice of materials available for the 3D printing of aligners is still reduced. The resin that has been most tested as an aligner material [4], the Dental LT Clear (Formlabs, Somerville, MA, USA), is actually meant for occlusal splint fabrication [5]. Of the few materials currently marketed for the direct printing of aligners [4,5], Tera Harz TC-85 DAC resin (Graphy, Seoul, Republic of Korea) has been recently tested for surface roughness in comparison with Invisalign aligners [6,7]. Koletsi et al. assessed the surface roughness of ‘as-received’ aligners and after 1 week of intraoral use [7]. It was reported that, for direct-printed aligners, roughness parameters increased significantly with function [7]. The observation that the aligners tested in the mentioned study had been cured according to an earlier protocol in the presence of oxygen prompted Eslami et al. to evaluate the properties of 3D printed aligners cured in a nitrogen chamber [6].

In addition to curing conditions, the orientation of printing layers has also been indicated as a possibly influential factor for the surface characteristics of additively manufactured aligners [2,3]. Yet no study has systematically addressed this issue. Generally speaking, no assessment of the surface gloss has, so far, been provided in the literature for direct-printed aligners.

Therefore, the present study is directed toward assessing the effect of build orientation on surface roughness and gloss of resins for printing orthodontic devices such as aligners and occlusal splints. The null hypothesis that no change in these properties occurs when the material is layered at different angulations relative to the print platform was placed under test.

## 2. Materials and Methods

**Figure 1:**
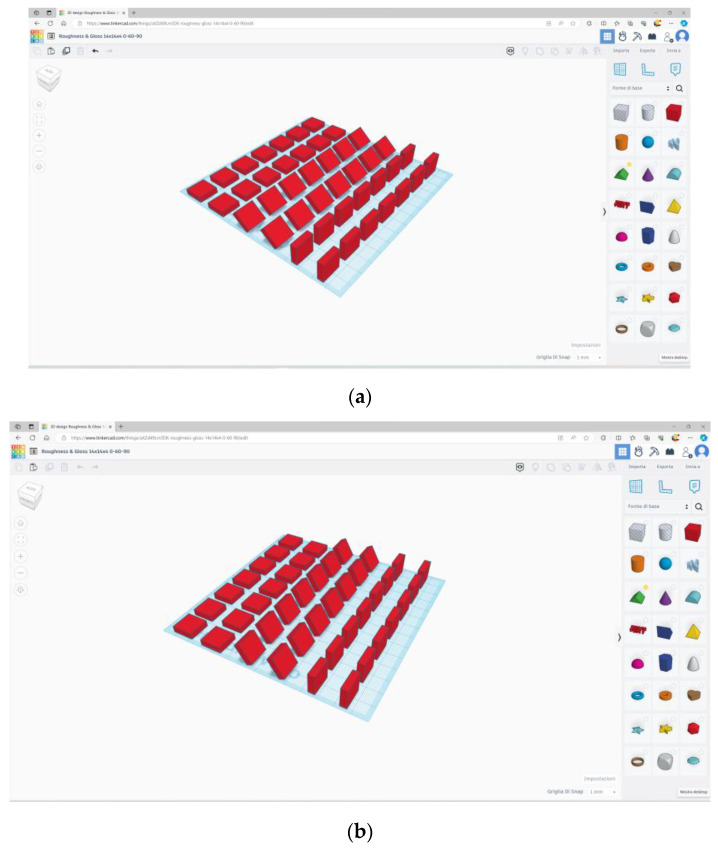
Design of specimens with the Tinkercad software. (**a**) Specimens to be printed with Dental LT Clear V2 resin, with angulations of 0°, 40°, and 90° to the building platform. (**b**) Specimens to be printed with Tera Harz TC-85 DAC resin, with angulations of 0°, 60°, and 90° to the building platform.

Square specimens of 14 × 14 mm and 4 mm in thickness were designed using the Tinkercad software (Autodesk, San Rafael, CA, USA; www.tinkercad.com accessed on 19 May 2024) (Figure 1) to be printed either with Dental LT Clear V2 resin (LT) or with Tera Harz TC-85 DAC (TC). Table 1 reports the chemical composition of the tested resins [8,9].

The specimens were designed with three different orientations relative to the printing platform: 0° (horizontal, H), 90° (vertical, V), and with the orientation recommended by the resin’s manufacturer (M), i.e., 40° for LT [10] and 60° for TC [11]. The experimental groups were, therefore, defined as follows: (TC-H) TC specimens printed horizontally; (TC-V) TC specimens printed vertically; (TC-M) TC specimens printed at the angulation recommended by the manufacturer; (LT-H) LT specimens printed horizontally; (LT-V) LT specimens printed vertically; (LT-M) LT specimens printed at the angulation recommended by the manufacturer. The projects were then exported in the .stl file format. Based on sample size calculations performed in previous studies with similar objectives [12,13], 10 specimens per experimental group were printed.

### 2.1. LT Specimens Printing and Post-Processing

For the manufacturing of LT specimens, the .stl file was imported into the PreForm software (Formlabs, Somerville, MA, USA; https://formlabs.com/it/software/preform/ accessed on 19 May 2024) for automatic support calculations and slicing. The printing layer thickness was set to 100 µm. Specimens were 3D printed with the Formlabs 3B 3D printer (Formlabs, Somerville, MA, USA). After printing, specimens were removed from the platform and, while still retaining raft and supports, they were subjected to washing to remove the uncured resin. Such a procedure was performed for 15 min by means of the FormWash automated washing machine (Formlabs, Somerville, MA, USA), using 99% isopropyl alcohol (IPA). After washing, the specimens were immersed for 5 min in fresh 99% IPA. Then, post-curing was performed for 20 min at 60°, using the proprietary device FormCure (Formlabs, Somerville, MA, USA). Subsequently, the supports were removed.

### 2.2. TC Specimens Printing and Post-Processing

To design the supports for the TC specimens, the .stl file was imported into the Uniz software, version 2.6.1.11 (Uniz, San Diego, CA, USA). Printing was performed with the Sonic XL 4K 2022 printer (Phrozen, Hsinchu, Taiwan) in layers of 100 µm in thickness. Thereafter, specimens were separated from the building plate and placed twice for 5 min in a centrifuge spinning at 1000× *g* revolutions per minute. Specimens were then dried with compressed air and the supports were removed. Post-polymerization was performed for 14 min in the absence of oxygen within a curing unit equipped with a nitrogen generator (Graphy Cure THC 2, Graphy Inc., Seoul, Republic of Korea). Following post-curing, the supports were removed, and specimens were washed in an ultrasonic cleaning machine filled with distilled water at 80° for 2 min. Subsequently, specimens were placed in boiling water at 100° for 1 min and, finally, dried with a drying machine for 5 min [14].

### 2.3. Surface Roughness Assessment

A profilometer (Mitutoyo SJ-201P, Mitutoyo, Kanagawa, Japan) set with a cutoff value of 0.8 mm, a stylus speed of 0.5 mm/s, and a tracking length of 5.0 mm was used to assess surface roughness (Ra). The measurement set up was standardized by means of a custom mold for both the handpiece of the instrument and the specimen [15]. Mean Ra (µm) was recorded.

### 2.4. Surface Gloss Assessment

Gloss was recorded using a small-area glossmeter (JND- XA6-SA; VTSYIQI Lab Measuring Instruments, Hafei, China) with a 2 mm × 2 mm square measuring area at a 60° angle [16]. A proprietary grey mold was utilized to eliminate the influence of room light and maintain the exact position of the specimen relative to the glossmeter reading area.

All surface roughness and gloss recordings were taken by the same operator who was experienced with the measuring devices and techniques (AV).

### 2.5. Statistical Analysis

Having checked that the collected data met the requirements of normality of data distribution (Shapiro–Wilk test) and homogeneity of group variances (Levene’s test), two separate two-way analyses of variance (ANOVAs) were applied to the surface roughness and gloss datasets. In each analysis, the optical property was considered as the dependent variable, while type of material and print direction were the factors. The Tukey’s test was used for post-hoc comparisons as needed. In all the tests, the level of significance was set to *p* < 0.05. The statistical calculations were handled by the PASW Statistics 18 software (SPSS Inc., Chicago, IL, USA).

## 3. Results

### 3.1. Surface Roughness

Table 2 reports the descriptive statistics of the roughness measurements. The two-way ANOVAs revealed that the material type did not significantly influence roughness (*p* = 0.08). Conversely, print direction was an influential factor (*p* < 0.001), and the post-hoc test disclosed that the print orientation recommended by the respective manufacturer yielded the roughest specimens (*p* < 0.05). Printing in the vertical direction resulted in higher roughness than in the horizontal direction, and also this difference was found to be statistically significant (*p* < 0.05). The material–print-direction interaction was not statistically significant (*p* = 0.12).

### 3.2. Surface Gloss

Table 3 reports the descriptive statistics of the gloss measurements. The two-way ANOVAs revealed that material type was a significant factor for surface gloss per se (*p* < 0.001), and, regardless of the print direction, TC exhibited significantly higher gloss than LT (*p* < 0.05). Also, print direction was found to be a significant factor per se (*p* < 0.001). According to the post-hoc test, regardless of the material, vertical printing resulted in significantly higher specimens’ gloss than printing in the other two directions (*p* < 0.05). The between-factor interaction was not statistically significant (*p* = 0.06).

## 4. Discussion

The performed roughness and gloss measurements were meant to investigate surface characteristics of 3D printed resins for aligners and occlusal splints that affect their esthetic properties and biofilm resistance.

Roughness can be quantified by different linear (Ra, Rq, Rz) or three-dimensional (Sa, Sq, Sz) variables.

The Ra parameter determined in the present study is the arithmetic average of the absolute values of the profile heights over the evaluation length [17]. Ra was selected because it provides an assessment of the average surface roughness [18].

Gloss quantifies the specular reflection from a surface [19].

It is computed by relating the amount of light reaching a surface at a 60° angle to the amount of light bouncing from the surface at an equal and opposite angle. Gloss is influenced by the optical characteristics of the material, particularly the refractive index, as well as by the surface morphology of the object. A coarser surface appears less glossy and, by reflecting a relatively greater amount of light back to the observer’s eye, it makes the orthodontic device visible in the mouth [2].

The data collected in the present investigation led to rejection of the formulated null hypothesis.

Concerning the surface roughness, print direction was found to be a significant factor regardless of the material. The smoothest surface was obtained when the resin was layered horizontally. This finding had been predicted in previous literature [2,3,20], and was attributed to the increased number of layers in vertical prints which create multiple surface steps.

Print angulation was not found to significantly influence the accuracy of the aligners printed with LT [21], as well as the mechanical properties of the aligners printed with TC [22]. Controversial information is present in the literature regarding the amount of time and material needed for vertical versus horizontal prints of aligners, although these issues have not been systematically addressed yet.

The manufacturers of the tested resins recommend printing the appliances obliquely, at an angle of 40° (LT) or 60° (TC) to the build platform. According to the manufacturer of TC, when printing at a 60° angle, resin tends to overflow in the incisors. Resin excess in this area is more easily removed with centrifugation than the diffuse build ups that develop in horizontally printed manufacts.

**Figure 2:**
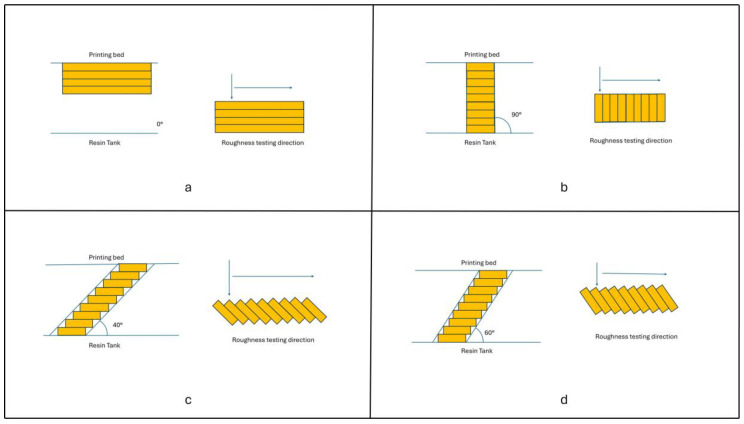
Diagrams illustrating the different tracks followed by the profilometer stylus when measuring surface roughness on specimens printed with different angulations to the printing bed: (**a**) 0°, horizontal; (**b**) 90°, vertical, (**c**) 40°, oblique according to Dental LT Clear V2 resin’s manufacturer, and (**d**) 60°, oblique according to TC-85 DAC resin’s manufacturer (Graphy, Seoul, Republic of Korea).

Interestingly, both materials exhibited the greatest roughness when printed obliquely. This finding can be ascribed to the printing geometry. In a horizontally printed object, the tracking length of the profilometer remains within the same printed layer (Figure 2a), while, in a vertically printed specimen, the stylus moves along a series of layers (Figure 2b) whose number depends on the printing resolution. Still, in a vertical print, the layers are levelled (Figure 2b). Conversely, in oblique printing, layers are not at the same level, yielding a staired surface that the profilometer detector steps through (Figure 2c,d).

The printing material did not emerge as an influential factor for roughness per se. The two tested resins differed not only in chemical composition, but also in the post-printing protocol. For TC, the procedure involved centrifugation, rather than immersion, in an organic solvent and curing in a nitrogen chamber that replaced oxygen. It has been stated that a deeper curing of the polymer, as obtained in nitrogen-saturated conditions, results in lower surface roughness of the printed manufact [23]. However, in the present study, no significant difference in Ra emerged between TC specimens post-cured in a nitrogen generator and LT specimens post-polymerized in an oxygen atmosphere. As for LT, it should be reiterated that, although it has been tested in previous research as an aligner material, it is actually marketed for printing occlusal splints. The manufacturer also recommends polishing the outer surface of the splint ‘with traditional polishing tools and materials commonly used for dental acrylics’ [10]. This treatment was omitted in the present study protocol as it is not indicated for aligners.

A direct comparison of the roughness measurements reported in the present study with data available in the literature is not feasible, as the two previous studies where TC has been tested recorded different surface parameters than Ra [6,7]. Regarding LT, no prior assessment of roughness has been provided.

In any case, the Ra values measured for 3D printed resins in the present study were significantly lower than those reported for thermoformed PET-G by Staderini et al. [17]. This finding is consistent with the results of the investigation by Eslami et al. [6], who compared TC aligners cured in a nitrogen chamber with Invisalign retainers.

Gloss resulted to be significantly influenced by both material type and print direction.

With either resin, vertical printing yielded the highest gloss, and the difference was statistically significant. A possible explanation for this finding is that the vertical print orientation minimized the gravity-driven collection of resin, which may render the aligner surface foggy.

When considering the resin irrespective of the build orientation, specimens printed with TC were significantly glossier. Centrifugal cleaning may have positively contributed to the glossier aspect of TC specimens. Mechanical cleaning by centrifugation has been reported to be more effective at removing uncured resin than chemical cleaning by immersion in IPA [24], with an additional benefit to esthetics. In contrast, the presence of residual uncured monomer would cause the resin to appear foggy [2,20].

The superiority of TC in gloss terms may also be ascribed to the advanced curing of the resin obtained in the nitrogen-containing chamber [14,23]. Vichi et al. assessed gloss and roughness of a 3D printed resin for permanent prosthetic restorations and reported that the specimens post-cured in a nitrogen chamber were significantly glossier than those finished and polished with different systems [13]. Another study indicated that post-curing in a nitrogen-saturated atmosphere significantly increased the resistance to discoloration to wine and curry of a resin marketed by Graphy for the 3D printing of permanent prosthodontic crowns [25].

A direct comparison of the collected gloss measurements with existing evidence is not feasible, as this relevant optical characteristic has been overlooked by previous research that has focused on other properties, such as transparency, translucency, and color stability [26,27].

As a possible limitation of the study, it can be mentioned that unused specimens were tested. It may, indeed, be meaningful to assess surface roughness and gloss of specimens that have been aged in the laboratory to simulate the effects of clinical service. Also, aligners retrieved after use could be subjected to surface roughness and gloss measurements; that, however, would require different equipment from that used in the present investigation, as readings should be performed on curved surfaces.

With regard to the stability of the optical properties, some concern has been raised that 3D printed resins may be relatively more prone to discoloration with time due to their greater susceptibility to absorbing water and pigmented solutions [28]. Nevertheless, although this issue is understandably relevant for 3D printed restorative materials, it can be downsized for aligners that are meant to be replaced after 7–10 days of clinical service, according to the current clinical protocols.

Based on this same observation, the concern regarding the increase in surface roughness and porosity reported by Eslami et al. [6] for aligners 3D printed with TC after 1 week of use can also be reconsidered.

## 5. Conclusions

Although LT is marketed as a material for occlusal splints, it has been utilized to 3D print aligners. The type of resin was not an influential factor for surface roughness per se. The finding of a higher roughness for vertically printed specimens in comparison with horizontally printed ones is in line with previous research and can be explained by the presence of a greater number of layers in vertical specimens. Horizontal printing produced the smoothest specimens. However, this print direction requires a larger surface on the building platform and may favor the development of resin build-ups in some areas of the aligner due to gravity. The Ra values measured for 3D printed resins in the present study were remarkably lower than those reported in the literature for thermoformed PET-G.

The superior gloss exhibited by TC regardless of the print angulation could be related to the effective cleaning of the uncured resin by centrifugation, as well as to a high degree of conversion of the monomers achieved by curing in a nitrogen atmosphere. No previous data are available for gloss for comparative purposes, as the present study is the first one to assess this property for aligner materials.

## Figures and Tables

**Table 1:** Chemical composition of the tested materials.

Name	Manufacturer	Chemical Composition
Tera Harz TC-85 DAC	Graphy, Seoul, Republic of Korea	GR30860 and GR3060 oligomers, bis(2,4,6-trimethylbenzoyl)-phenylphosphine oxide (Irgacure 819, BASF SE, Ludwigshafen, Germany)
Dental LT Clear	Formlabs, Somerville, MA, USA	Bisphenol A dimethacrylate (50–70 wt%, 2-hydroxyethyl methacrylate, 7–10 wt%, urethane dimethacrylate 25–45 wt%) [9]

**Table 2:** Descriptive statistics of surface roughness measurements (Ra, µm). Different superscript letters highlight statistically significant differences in roughness among print directions regardless of the material type, according to the post-hoc test (*p* < 0.05).

Material	Printing Orientation	N	Mean ± Standard Deviation
LT Clear V2	Horizontal	10	1.36 ± 0.14
	Manufacturer’s instructions	10	4.86 ± 0.21
	Vertical	10	1.73 ± 0.10
	Total	30	2.65 ±1.60
TC-85 DAC	Horizontal	10	1.39 ± 0.13
	Manufacturer’s instructions	10	4.79 ± 0.26
	Vertical	10	1.49 ± 0.32
	Total	30	2.55 ± 1.62
Print direction	Horizontal ^a^	20	1.38 ± 0.13
	Manufacturer’s instructions ^c^	20	4.82 ± 0.23
	Vertical ^b^	20	1.61 ± 0.26

**Table 3:** Descriptive statistics of surface gloss measurements (GU). Different superscript capital letters mark the significant difference between the materials regardless of the print direction. Different superscript lowercase letters label statistically significant differences in gloss among print directions regardless of the material type, according to the post-hoc test (*p* < 0.05).

Material	Printing Orientation	N	Mean ± Standard Deviation
LT Clear V2 ^B^	Horizontal	10	1.95 ± 0.15
	Manufacturer’s instruction	10	2.05 ± 0.15
	Vertical	10	5.50 ± 1.22
	Total	30	3.16 ± 1.81
TC-85 DAC ^A^	Horizontal	10	4.05 ± 0.43
	Manufacturer’s instruction	10	2.80 ± 0.48
	Vertical	10	7.00 ± 1.63
	Total	30	4.61 ± 2.04
Print direction	Horizontal ^b^	20	3.00 ± 1.12
	Manufacturer’s instruction ^b^	20	2.42 ± 0.51
	Vertical ^a^	20	6.25 ± 1.61

## Data Availability

The data presented in this study are available on request from the corresponding author. The data are not publicly available due to university policy on access.
